# Vanillin Cross-Linked Chitosan Film with Controlled Release of Green Tea Polyphenols for Active Food Packaging

**DOI:** 10.1021/acsfoodscitech.3c00222

**Published:** 2023-10-09

**Authors:** Jessica R. Westlake, Maisem Laabei, Yunhong Jiang, Wen Chyin Yew, Darren L. Smith, Andrew D. Burrows, Ming Xie

**Affiliations:** † Department of Chemical Engineering, University of Bath, Bath BA2 7AY, U.K.; ‡ Department of Chemistry, University of Bath, Bath BA2 7AY, U.K.; ◊ Department of Biology, University of Bath, Bath BA2 7AY, U.K.; ¥ Department of Applied Sciences, Northumbria University, Newcastle NE7 7XA, U.K.

**Keywords:** active packaging, chitosan, antimicrobial, controlled release, biodegradation, waste valorization

## Abstract

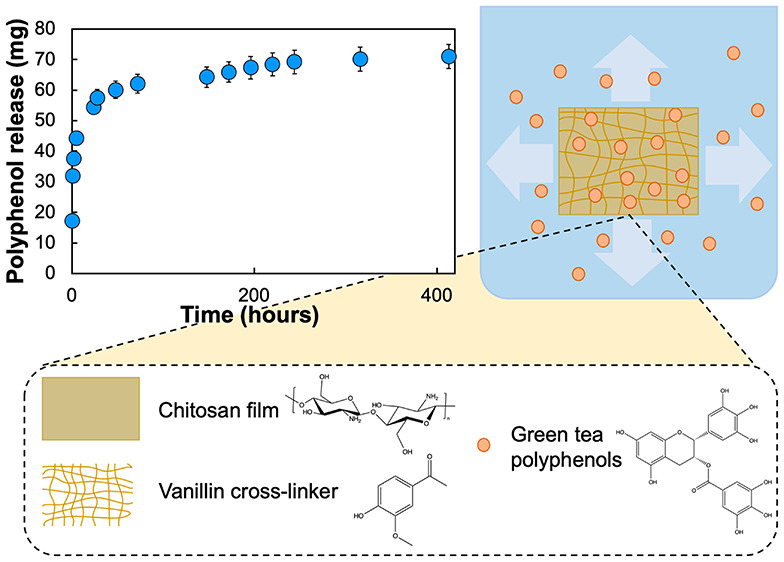

We report a novel cross-linked chitosan composite film containing vanillin, glycerol, and green tea extract. The effects of vanillin-mediated cross-linking and the incorporation of antimicrobial green tea polyphenols were investigated. The cross-linking effect, confirmed by Fourier transform infrared (FTIR) analysis, increased the tensile strength of the biopolymer film to 20.9 ± 3 MPa. The release kinetics of polyphenols from the chitosan–vanillin matrix was studied, and we reported an initial burst release (8 h) followed by controlled release (8 to 400 h). It was found that both vanillin and green tea polyphenols were successful inhibitors of foodborne bacteria, with a minimum inhibitory concentration of the tea polyphenols determined as 0.15 mg/mL (*Staphylococcus aureus*). These active components also displayed strong antioxidant capacities, with polyphenols quenching >80% of 2,2-diphenyl-1-picrylhydrazyl (DPPH) radicals at all concentrations tested. Degradation results revealed that there was a significant (>85%) mass loss of all samples after being buried in compost for 12 weeks. The biopolymeric films, prepared by solvent casting methods, adhere to green chemistry and waste valorization principles. The one-pot recipe reported may also be applied to other cross-linkers and active compounds with similar chemical functionalities. Based on the obtained results, the presented material provides a promising starting point for the development of a degradable active packaging material.

## 1 Introduction

The emergence of biodegradable active packaging has unlocked the potential to simultaneously tackle two key environmental issues: food waste and plastic waste. The food packaging industry is the third largest industry globally and is the largest contributor to municipal solid waste.^1−3^ UNEP recently estimated that food waste amounted to over 900 million tonnes in 2019, which corresponded to almost 20% of total food production.^4^ Currently, there is significant societal pressure stemming from environmental damage caused by conventional plastics. This has initiated a movement toward the use of biobased and degradable polymers. In recent years, governing bodies have begun to implement more strict policies relating to conventional fossil-fuel-derived plastics and related pollution, paving the way for more sustainable alternatives.^5^

Active packaging materials may incorporate antimicrobial or antioxidant compounds into the packaging material itself. These materials aim to tackle issues relating to food spoilage, including the greenhouse gas (GHG) emissions released by food waste and the occurrence of foodborne diseases. Active packaging can effectively reduce the aforementioned issues by delaying lipid oxidation and microbial growth, hence preserving perishable foods.^6^ Controlled release technology has become a recent spotlight for active packaging research.^7−9^ Controlled release packaging has the potential to maximize the freshness of food by extending the release of antimicrobial compounds over time. Techniques used to achieve controlled release from films include cross-linking, chemical modification of polymers, multilayer film formation, and various encapsulation methods including the use of microparticles, nanoparticles, MOFs, COFs, or cyclodextrins.^10−13^ Although controlled release strategies are regularly reported, little attention is paid to the release time scale. Literature studies report varying time scales of release from under 24 h to over 140 h.^11,12,14^ Importantly, the time scale of the release study should emulate the time scale of food storage within the packaging material. Furthermore, the quantification of release into the packaging headspace under various conditions is crucial to avoid potential storage instabilities and to adhere to stringent safety legislation for commercialization.

Previous studies on active packaging have primarily concentrated on the use of traditional polymers or blends between biopolymers and fossil-fuel-derived polymers.^15−17^ However, a growing body of literature has focused on the use of naturally derived biopolymers to enhance the sustainability of these materials. The use of waste valorization and degradable biopolymers is becoming more popular in the literature as policies move toward circular economy targets.^18−20^ Chitosan is a highly abundant biopolymer produced by the deacetylation of chitin, a compound that may be extracted from crustacean shells, insect exoskeletons, or fungi cell walls.^21,22^ Chitosan has been extensively researched for use in active packaging due to its nontoxicity and inherent antimicrobial activity. Indeed, chitosan is approved as a food ingredient by the Food and Drug Administration (FDA). Current studies often focus on the use of composites of chitosan with other polymers, additives, and plasticizers. These composites are used to overcome problems common to many biopolymers including brittleness, low tensile properties, poor barrier properties, and low thermal stability values.^2,23,24^

Chitosan has a strong tendency for hydrogel formation. Therefore, cross-linking is often used to reduce the swelling index, overcome poor mechanical and barrier properties, and enhance thermal stability.^25^ So far, the cross-linking of chitosan has been most popular with compounds such as glutaraldehyde and citric acid, with only a few studies utilizing vanillin.^26−30^ Recently, there has been a movement toward the use of naturally derived cross-linkers. For example, quercetin from onion food waste has been used in a number of studies to cross-link chitosan.^31^ Vanillin can be used in a similar way due to its aldehyde moiety, which is capable of forming a Schiff base interaction with the amino groups of chitosan.^32^ Tomadoni et al. recorded the optimization of vanillin–chitosan–glycerol film formulations by response surface methodology, comparing different film properties including mechanical, antioxidant, and barrier properties.^33^ Similarly, Zhang et al. reported the enhancement of the mechanical properties of chitosan by vanillin-mediated cross-linking.^34^ Vanillin can also impart antimicrobial effects to the active packaging material.^35,36^ To this end, Eelager et al. used vanillic acid in a chitosan–PVA blend, affording excellent antimicrobial activity against various food-related bacteria.^32^ However, the aroma of vanillin has the potential to alter the organoleptic properties of food. Therefore, it is beneficial to determine and minimize the migration of vanillin from the packaging material.

Similarly, active packaging research is moving toward the replacement of potentially harmful synthetic antioxidants such as butylated hydroxyanisole (BHA) and butylated hydroxytoluene (BHT) with natural antimicrobial compounds. Many studies report the use of metal nanomaterials such as silver as antimicrobial compounds or additives.^37,38^ However, these materials have been shown to be toxic to cells, and therefore, rigorous safety experiments would be required for commercialization. Hence, the use of natural components may reduce barriers to commercialization, as many compounds are already generally recognized as safe (GRAS). Santhosh et al. recently discussed the benefits of using natural antimicrobials, including polyphenols from plant byproducts for packaging materials.^39^ Indeed, essential oils (EOs) are commonly used in the literature in combination with chitosan to form active films with high antioxidant and antimicrobial properties.^36,40,41^ However, the sensory quality of food can often be altered due to the strong scent and flavor of EOs, leading to potential consumer nonacceptance.^36,42^ Green tea extract has been used in active packaging materials in various studies both as an active agent and as a cross-linker.^43−45^ Green tea extract contains gallic acid, epigallocatechin gallate, and seven other major catechins, which are responsible for its antioxidant and antimicrobial properties.^7^ Green tea is an inexpensive, readily available material with many low-intensity extraction methods.^46^ Moreover, antioxidant polyphenol compounds may be extracted from agricultural waste or waste tea leaves.^47−52^

The aim of this study was to design, fabricate, and analyze cross-linked chitosan films for active packaging using a facile, one-pot formulation and solvent casting techniques. The primary objective was to determine the release profile of polyphenols entrapped within the biopolymer film using UV–visible spectroscopy. Furthermore, we used a suite of analytical characterization techniques to determine the physical, mechanical, antimicrobial, and degradation properties of the films. Overall, our results indicate a promising starting point for the development of a degradable active packaging material.

## 2 Materials and Methods

### 2.1 Chemicals

High-molecular-weight (HM_W_) chitosan (310–375 kDa, >75% degree of deacetylation), polyphenon 60 extract from green tea, vanillin, 2,2-diphenyl-1-picrylhydrazyl (DPPH) radical, glycerol, and ethanol were purchased from Sigma-Aldrich. Reagent-grade glacial acetic acid was obtained from Alfa Aesar. Deionized water was used to prepare all aqueous solutions.

### 2.2 Preparation of CVGP Active Films

The synthetic method was adapted from a method described by Tomadoni et al.^33^ 2% (w/v) chitosan (HM_W_) was dissolved in 1% aq acetic acid at room temperature (RT) under magnetic stirring with 45% (w/w) glycerol and 37.5% (w/w) vanillin. After 18 h, 20% (w/w) green tea extract was added. After 3 h, the solution was filtered to 100 μm under vacuum using a nylon net filter to remove impurities. The solution was then degassed and cast onto a glass plate. The film was left to evaporate solvent for 48–72 h at RT to afford a transparent yellow film, which was denoted CVGP. For comparisons, non-cross-linked films were denoted CG, and films without polyphenols were denoted CVG.

### 2.3 Hot Pressing Films

A digital heat press machine (Display4top, U.K.) was used at 160 °C to hot-press films for 2–5 min. Films were allowed to cool to RT before characterization.

### 2.4 Characterization of CVGP Films

Prior to any characterization and between analyses, all films were equilibrated in a desiccator at 25% RH and 25 ± 1 °C. At least five samples of each film were prepared, and selected analyses were carried out on films from different experimental series. Laboratory conditions were 25 ± 1 °C and 35% RH, and fridge storage was carried out at 4 ± 1 °C and 25% RH. The thickness of each film was measured using a digital micrometer (Digi-Micrometer, Fowler Precision) with a precision of ±0.5 μm. An average was calculated using eight measurements of thickness, and the standard deviation of each value was calculated. The average density of CVGP films was calculated by dividing the mass of the film by the volume of the film. The density of CVGP films was determined as 0.26 g cm^–3^.

#### 2.4.1 Film Microstructure

The film microstructure was characterized using field emission scanning electron microscopy (Jeol 7900, Japan) with 5 kV acceleration voltage under high vacuum. The samples were mounted on aluminum stubs with double-sided carbon tape and placed into a vacuum chamber for 18 h before imaging. Cross-sectional images were obtained by freeze-fracturing samples in liquid nitrogen prior to mounting. Magnification levels used ranged from 1000 to 10,000×.

#### 2.4.2 Color Measurements

The L* (lightness/darkness), a* (redness/greenness), and b* (yellowness/blueness) values of the surface of CVGP and CVG films were measured using a colorimeter with an enclosed illumination cube (VeriVide, DigiEye, U.K.) against a white calibration plate as the background. A standard calibrated lighting environment (CIE) was used with a characterized digital camera for observation. DigiPix software was used for the color measurement of the films imaged. Each film was measured at a minimum of eight stochastic points to calculate the average and standard deviation. The yellowness index (YI) was calculated using the following equation.
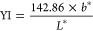
1

#### 2.4.3 Mechanical Properties

A texture analyzer (INSTRON 3369) with a 100 N load cell was used to measure the tensile properties of chitosan films according to the ASTM D882 method.^53^ Pneumatic grips were used to clamp the films using a pressure of 4 bar. The initial grip separation and velocity were adjusted to 100 mm and 12.5 mm min^–1^, respectively. The values of force and distance were recorded during the extension of the biopolymer film strips. Between 5 and 12 samples of each film were analyzed to calculate the average and standard deviation. The elongation at break, tensile strength, Young’s modulus, and force at break values were determined for each sample.

#### 2.4.4 Water Contact Angle Measurement (θ)

Contact angle measurements were made using the sessile drop method in air at RT. Droplets of water (5 μL) were deposited on the horizontal film surface with a precision syringe using an OCA 25 system (DataPhysics, Germany) equipped with SCA 20 module base software. Still images were obtained for calculation of the contact angle, and four measurements were taken for each sample to calculate the average contact angle and standard deviation.

#### 2.4.5 Moisture Content Analysis

For the determination of the moisture content (MC), film samples were cut into square shapes with dimensions of 4 cm × 4 cm, weighed, and dried in an air-circulating oven (Lincat, U.K.) at 105 ± 2 °C until they reached a constant weight. The values of MC were calculated using the following equation; at least three measurements were taken to calculate the average and standard deviation.
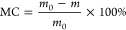
2where *m*_0_ is the initial mass and *m* is the final mass.

#### 2.4.6 Fourier Transform Infrared (FTIR) Spectroscopy

A PerkinElmer frontier Fourier transform infrared spectrometer (PerkinElmer) was used to record the spectra of the dried films and individual film components. The spectral resolution was 4 cm^–1^, and 32 scans were acquired for each spectrum (4000–400 cm^–1^). The FTIR spectra of the samples were acquired directly.

#### 2.4.7 Dynamic Mechanical Analysis (DMA)

The mechanical properties of chitosan composite films were measured using a DMA 1 STAR SYSTEM instrument (Mettler Toledo, U.K.), using a tension clamp for rectangular films and a temperature sweeping from −5 to 180 °C, under the following conditions: a heating ramp at 10 °C per minute, a preload force of 1.5 N, and a frequency of 1 Hz.

#### 2.4.8 Thermogravimetric Analysis (TGA)

TGA measurements were carried out on a Setsys Evolution TGA 16/18 thermogravimetric analyzer (Setaram, Switzerland). Thermal degradation was performed in an atmosphere of air up to 500 °C with a heating ramp of 10 °C per minute. Samples of 15–20 mg were used. Weight loss calculations were carried out for each step of degradation, and the moisture content was evaluated from the mass loss via moisture evaporation at 105 °C.

### 2.5 Release Kinetics

Samples of chitosan composite films were cut to 4 × 4 cm and submerged in a food simulant of 10 mL of 50% (v/v) ethanol in water in closed containers. A UV–vis spectrometer (Agilent Technologies) was used to determine the concentration of polyphenols (275 nm) and vanillin (312 nm) released into an aliquot of food simulant at regular time intervals up to >400 h. After each reading, the film was removed from the simulant and placed into the same volume of fresh simulant to allow for a cumulative reading. All readings were taken in triplicate. Calibration curves of vanillin and polyphenols were recorded and used to calculate the amount of each compound released over time (Figure S1 in the Supporting Information).

The release kinetics of polyphenols from the chitosan–vanillin matrix were initially fit to zero- and first-order kinetics (eqs 3 and 4). The kinetics were better described using the Korsmeyer–Peppas model (eq 5), the Higuchi model (eq 6), and the approximation derived from Fick’s second law (eq 7). For both eqs 5 and 7, the approximation can only be used for values of *M_t_*/*M*_∞_ ≤ 2/3. For eq 7, the plot of *M_t_*/*M*_∞_ versus *t*^1/2^ results in a linear curve with slope *k* (eq 6).^7,54^ The diffusion coefficient (*D*) for burst release was calculated by rearranging eq 8. The kinetic constants were determined in each case, and the *R*^2^ values of each mathematical fitting were compared.
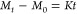
3
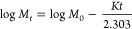
4
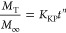
5
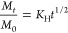
6
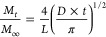
7
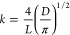
8where *M*_*t*_ is the mass of the active compound at time *t* in hours, *M*_*∞*_ is the mass at *t* = *∞*, *k* is the kinetic constant for zero- or first-order kinetics, *K*_KP_ is the kinetic constant for the Korsmeyer–Peppas (KP) model, *n* is the diffusion or release exponent for the KP model, *M*_*t*_/*M*_∞_ is the fraction of drug released at time *t*, *K*_H_ is the Higuchi kinetic constant, *L* is the thickness of the film in cm, and *D* is the diffusion coefficient in m^2^ s^–1^.

### 2.6 Antimicrobial and Antioxidant Testing of CVGP Films

#### 2.6.1 Bacterial Culture Conditions and Minimum Inhibitory Concentration Determination

*Staphylococcus aureus* strain SH1000 was grown on tryptic soy agar at 37 °C for 18 h. *Escherichia coli* strain K12 was grown on Luria–Bertani agar at 37 °C for 18 h. The antibacterial activity of viscous film-forming solutions (CG, CVG, and CVGP) and the minimal inhibitory concentration (MIC) of green tea polyphenols against bacterial strains listed above were achieved using modified versions of the agar diffusion and broth microdilution methods, as described by the Clinical and Laboratory Standards Institute.^55^ Briefly, pure colonies of *S. aureus* or *E. coli* were used to inoculate separate 12 mL polystyrene test tubes containing Mueller–Hinton broth (MHB), and cultures were incubated for 18 h at 37 °C while being shaken at 180 rpm. Overnight cultures were subsequently diluted 1:100 in fresh MHB and grown at 37 °C with shaking at 180 rpm to achieve an absorbance (OD600 nm) of 0.5–0.6, indicating the exponential phase of growth. Aliquots of 0.5 McFarland standardized inoculum of bacteria were calculated, and approximately 5 × 105 CFUmL^–1^ was spread on Mueller–Hinton agar to use in the agar diffusion assay. Aliquots of samples (50 μL) were dropped onto agar bacterial lawn plates and incubated for 18 h at 37 °C. The antimicrobial activity of compounds was indicated by the presence of zones of inhibition.

The growth dynamics of bacteria challenged with the compounds listed above were examined using the broth microdilution method. Here, compounds were diluted in MHB and pipetted into sterile polystyrene 96 well round-bottom microtiter plates to a final concentration range of 0–5 mg mL^–1^. Bacterial cultures were grown over 18 h at 37 °C with shaking at 180 rpm in a microtiter plate reader, where the absorbance (OD600 nm) of individual wells containing cultures and active compounds was measured every 10 min. For the determination of the inhibitory effect of individual film components, the growth dynamics experiment was repeated with compounds diluted to 20 mg mL^–1^. For this experiment, a different strain of *S. aureus* (DSMZ 20331) was used with Luria–Bertani (LB) broth.

#### 2.6.2 Antiviral Test

The antiviral efficacy of biopolymer films was determined by following a modified protocol.^56^ Briefly, each film was placed in individual wells on a 6-well plate. A total of 10 μL of Phage Phi6 (DSM 21518) stock in Lysogeny broth (LB media and 0.01 M CaCl_2_) was added to the surface and immobilized across the slide by addition of a coverslip. This was then incubated for 1 h at room temperature (20–25 °C). The samples were submerged in 1 mL of LB buffer, the coverslip was removed, and surfaces were washed with gentle pipetting. Phi6 submerged in LB was subject to a 10-fold serial dilution. A phage overlay plate was created by the lower layer LB buffer with 1.5% w/v Difco Bacterial Agar, overlaid with LB with 0.4% w/v Difco agar, containing 200 μL of *Pseudomonas syringae* (DSMZ 21482) with a culture optical density of 0.5–0.7. A total of 10 μL of each dilution was added to the bacterial overlay, allowed to dry, and incubated for 18 h at room temperature (20–25 °C) prior to the plaque count.

#### 2.6.3 DPPH Antioxidant Activity Test

The antioxidant activities of polyphenols and vanillin were measured by using the 2,2-diphenyl-1-picrylhydrazyl (DPPH) radical method. Briefly, 0.75 mL of the sample extract or ethanol (blank) was mixed with 0.75 mL of ethanolic DPPH solution (0.784 mg mL^–1^). The mixtures were shaken and allowed to stand in the dark for 30 min at RT. The decrease in absorbance at 517 nm was then measured for each sample relative to the blank using a Cary-100 UV–visible spectrometer (Agilent Technologies). The DPPH radical scavenging activity was expressed as a percentage of radical scavenging capacity according to eq 9.
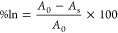
9where %In is the percentage of DPPH radical inhibition, *A*_0_ is the absorbance of the blank sample, and *A*_s_ is the absorbance of the sample.

### 2.7 Degradation Studies

Samples of CVGP and CG films were weighed to determine their initial mass and then buried in a container with either compost (Miracle Gro Peat Free) or soil obtained from campus (University of Bath, U.K.). In each case, the samples were stored at 80 ± 5% RH and 24 ± 2 °C, and the mass of each film was determined at regular intervals for up to 12 weeks. The initial degradability in seawater was determined for CVGP films using 100 mL of seawater obtained from Devon, U.K. Dry films were submerged in beakers containing seawater, and the remaining mass was measured after rinsing and drying the samples at regular intervals up to 8 weeks.

### 2.8 Statistical Analysis

Experimental data obtained were presented as mean values and the corresponding standard deviation (SD). Post hoc tests were carried out using the *t*-test with a one-tailed distribution. In all analyses, differences were accepted as significant when *p* < 0.05.

## 3 Results and Discussion

### 3.1 Key Physical and Chemical Properties of Cross-Linked Chitosan Films

#### 3.1.1 Appearance and Formulation

The RSM-optimized formulation proposed by Tomadoni et al. was modified to include active green tea polyphenols at a content of 20% (w/w of chitosan) to afford the biopolymeric film denoted “CVGP”.^33^ We report films synthesized using 2% (w/v) solutions of high-molecular-weight (310–375 kDa) chitosan with a deacetylation degree of >75% and a drying temperature of 25 °C. Similar formulations reported in the literature include a 1.5% (w/v) solution of chitosan with a reaction temperature of 72 °C^57^ and a 1% (w/v) solution at RT with a vanillin content of 20% (w/w).^58^ In the presented film, a cross-linking reaction arises due to the formation of a Schiff base between functional groups of chitosan and vanillin (Figure S2 in the Supporting Information). It is proposed that this cross-linking enhances the structure of the polymeric network, reducing water sensitivity and increasing tensile strength. It has been suggested that polyphenol–chitosan interactions arise in similar materials in the literature.^59−61^ Moreover, various studies have reported that tea polyphenols are capable of cross-linking chitosan.^62−64^ However, the properties of tea polyphenol cross-linked films have been reported to reduce drastically during storage due to facile oxidation.^7^ Therefore, the polyphenols were added to the film-forming solution at the end of the synthesis to maximize vanillin-mediated cross-linking and therefore optimize film properties.

#### 3.1.2 Chemical Structure

**Figure 1:**
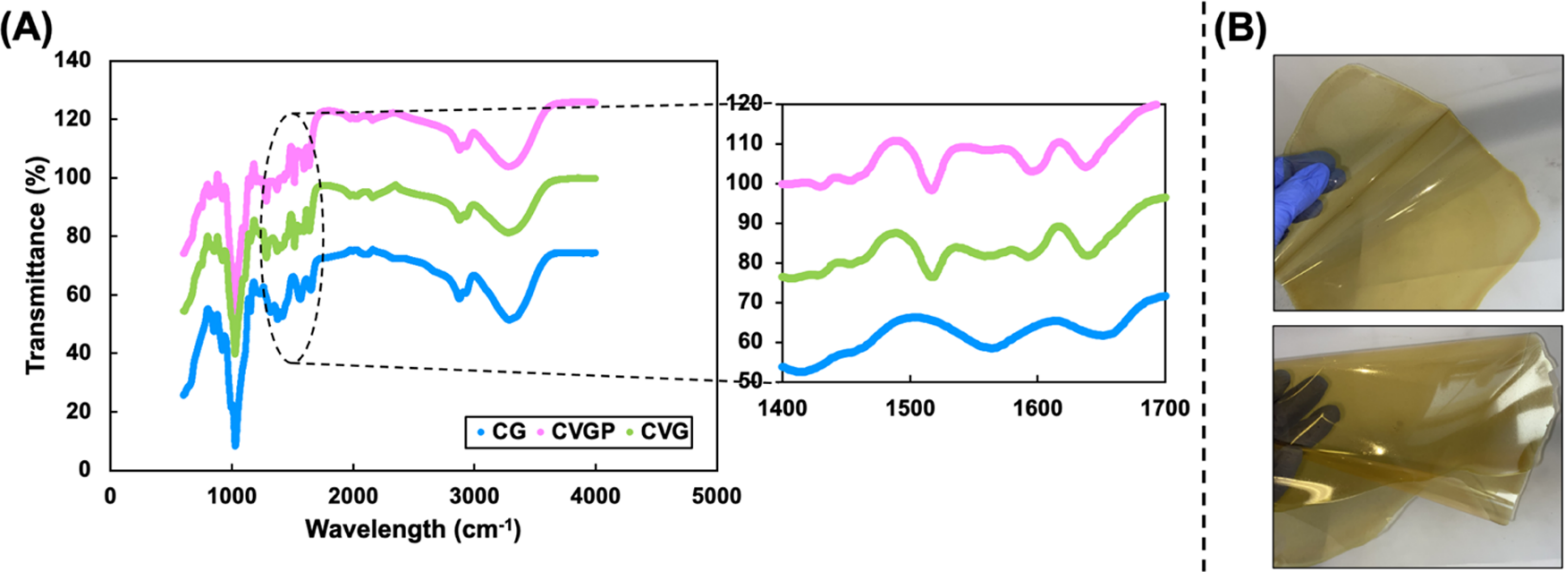
(A) FTIR spectra of chitosan-based biopolymer films (CG, CVG, and CVGP), with the section from 1400 to 1700 cm^–1^ enhanced for distinguishing key peaks including the Schiff base peak. (B) Images of a transparent yellow CVGP film.

FTIR analysis was performed to investigate the intermolecular interactions of the chitosan–vanillin-green tea matrix (Figure 1). The effect of cross-linking chitosan with vanillin was observed within the region from 1500 to 1700 cm^–1^. Consistent with the works of Zhang and Tomadoni, we observed a shift in the vibration of a peak in the carbonyl region from 1656 to 1644 cm^–1^ when comparing CG and CVG, indicating the formation of a Schiff base.^33,34^ The broad peak at 1567 cm^–1^ (NH bending in secondary amines) in CG is observed less intensely in CVG and CVGP, indicating that this functional group is involved in cross-linking and suggesting that most amine groups are involved in this interaction. The broad peak at 1656 cm^–1^ (C=O stretching in secondary amines) in CG does not appear in the cross-linked films. Instead, there is a sharp peak at 1644 cm^–1^ corresponding to the vibration of an imine band (C=N). Furthermore, vanillin cross-linked films produced a shark peak at 1518 cm^–1^ (–CO_2_ symmetric stretching) and a peak at 1598 cm^–1^ (C=C stretching in cyclic alkenes), not present in the CG film. Overall, the sharp peaks indicate an increase in the strength of intermolecular interactions in the cross-linked films. Peaks common to all films include C–O stretching vibrations at 1032 cm^–1^, symmetrical C–H stretching at 2887 cm^–1^, asymmetric C–H stretching (aliphatic CH_2_) at 2934 cm^–1^, and the broad peak at 3352 cm^–1^, indicating intermolecular hydrogen-bonded OH stretching alongside NH stretching (secondary amines), consistent with the results reported by Tomadoni et al.^33^

#### 3.1.3 Optical Properties of Chitosan Films

Color is an important parameter for consumer acceptance. However, many biopolymers or composites do not form colorless, transparent films. Table 1 shows the results from a colorimeter experiment on CVGP, CVG, and hot-pressed CVGP films. The L* (lightness/darkness), a* (redness/greenness), and b* (yellowness/blueness) values of the surface of the biopolymeric films are reported, and the yellowness index (YI) was calculated in each case using eq 1. Consistent with literature reports, cross-linking had a significant effect on optical film properties.^33^ The chitosan biopolymer films appeared yellow in color and were visually transparent. Other authors have reported similar observations, attributing the yellow appearance to the Schiff base formation^65,66^ or chemical modifications to the films.^67^ The results in Table 1 correlate well with previously published results by Zhang et al. for their chitosan film with a vanillin content of 50% (L* = 56.85 ± 0.67, a* = 6.87 ± 0.85, b* = 32.25 ± 0.41).^34^ The pristine chitosan film reported by the group had a lower b* value than the cross-linked film, indicating that vanillin cross-linking increased the yellowness of the film.^34^ In our study, the color of hot-pressed CVGP films was also investigated. It was concluded that the hot-pressed films were darker in color. This color change from yellow to brown may be attributed to the oxidation of green tea carotenoids at high temperatures during the hot-press process. While darker films may offer advantages when packaging light-sensitive foods, consumers are likely to prefer films similar in color to those of conventional, colorless packaging materials.

**Table 1:** Color Data Extracted from a Colorimeter Experiment on Various Chitosan Films, Where YI is the Yellowness Index and Superscript Letters Show the Statistical Significance of the Values

biopolymer film	YI	L*	a*	b*
CVGP hot pressed	79.9	57.0 ± 0.2^a^	22.4 ± 0.15^a^	31.9 ± 0.4^a^
CVG	45.2	97.2 ± 0.1^b^	–7.2 ± 0.1^b^	30.8 ± 0.1^b^
CVGP	67.4	88.2 ± 0.4^c^	2.1 ± 0.1^c^	41.6 ± 0.5^c^

The color change of CVGP films was investigated over a period of two months in different environments with color measurements at various intervals. In an open-air environment, the color began to darken from yellow to brown after 2 weeks. The total color change after two months was indicated by a reduction in the L* value from 81.9 ± 0.4 to 39.2 ± 0.3, an increase in the a* value, and a decrease in the b* value. In a fridge environment, the same color change began after 3 weeks. In a desiccator, the color was stable for at least two months. It was postulated that this color change was due to polyphenol or carotenoid oxidation. However, we noted that films of CVG also darkened to brown with extended storage in open-air environments, indicating that a different mechanism could also operate. Kraskouski et al. indicated that the browning of chitosan was due to the formation of melanoidins, which formed in their experiment due to the simultaneous hydrolysis and Maillard self-reaction of chitosan.^68^

### 3.2 Representative Mechanical and Surface Properties

#### 3.2.1 Mechanical Properties

The mechanical properties of biopolymer composites are related to their potential success as packaging materials. It is important that the materials are strong and extensible such that they can withstand consumer stresses and provide the protection of food during manufacturing, distribution, transportation, and storage. Important metrics include tensile strength, elongation at break, and Young’s modulus. The tensile strength of polymer films represents the maximum stress that the film can endure before breaking, and Young’s modulus (YM) is a measure of the stiffness of the film. The elongation at break relates to the extendibility of a polymer film and thus the ability of the material to wrap around food. We compared the mechanical properties of CG, CVG, and CVGP films. We also tested CVGP films with fridge storage and hot-press pretreatment. The material properties of chitosan depend strongly on the molecular weight and deacetylation degree of the polymer. Additionally, higher drying temperatures may increase the tensile strength by increasing the cohesive strength between polymer chains and reducing the free volume of the polymer matrix.^69^ Overall, the mechanical tests investigated the effect of adding GTE to the chitosan film and the effect of heat treatments on the film (Table 2).

**Table 2:** Mechanical Properties of Chitosan-Based Biopolymeric Films, Superscript Letters Show Significant Differences between the Values

biopolymer film	elongation at break (%)	thickness (μm)	tensile strength (MPa)	force at break (N)
CVGP hot pressed (HP)	3.4 ± 0.6^a^	70 ± 3.6^a^	17.9 ± 1.2^a^	22.0 ± 2.4^a^
CG	4.6 ± 0.04^b^	83 ± 4.7^b^	7.1 ± 0.1^b^	5.7 ± 0.1^b^
CVGP fridge (F)	4.4 ± 0.3^b^	65 ± 5.1^a^	29 ± 1.6^c^	25.9 ± 2.1^a^
CVG	3.1 ± 0.8^a^	68 ± 6.4^a^	12.1 ± 0.7^d^	12.9 ± 0.5^c^
CVGP	6.0 ± 0.5^c^	73 ± 6.4^a,b^	20.9 ± 2.9^a^	12.8 ± 0.9^c^

The results indicate that the tensile strength increased to 20.9 ± 2.9 MPa with vanillin-mediated cross-linking. Similarly, one study reported a 50% increase in the tensile strength of chitosan with vanillin-mediated cross-linking with a 0.5–10% vanillin content.^34^ The cross-linking of chitosan–CMC composites with glutaraldehyde has also been reported to increase the tensile strength of polymer films from 9.1 to 27.7 MPa.^27^ The group also reported that the incorporation of cinnamon essential oil reduced the tensile strength to 10.8 MPa.^27^ Contrastingly, in our study, we found that active agent incorporation improved the mechanical properties. The increase in the tensile strength between CVG and CVGP suggests that the polyphenols increased the strength of intermolecular and intramolecular forces within the polymer matrix. We postulate that the polyphenols may also cross-link the remaining chitosan functional groups. Additionally, polyphenol compounds have multiple hydrogen bonding sites that may interact with other polar groups within the chitosan–vanillin matrix. Furthermore, we concluded that refrigerator storage increased the tensile strength of the CVGP film due to a reduction in chain mobility. However, hot-press pretreatment did not significantly alter the tensile strength but reduced the EAB values and increased the brittleness of the film.

Tomadoni et al. reported that the drying temperature, vanillin content, and glycerol content all affected the YM value.^33^ The group reported a YM value for their optimum CVG formulation as 1225 ± 135 MPa.^33^ This value correlates well with the YM determined for our CVGP film as 1242 ± 62 MPa. However, the YM value for our CVG film was lower than that in the literature study at 945.3 ± 96 MPa. This discrepancy may be attributed to the lower drying temperature used in our study. Another report, using vanillin as the antimicrobial agent rather than the cross-linker for chitosan, reported a tensile strength value of 31 MPa with a drying temperature of 40 °C.^70^ Conventional plastics used in food packaging such as polycaprolactone (PCL) and low-density polyethylene (LDPE) have tensile strength values in the region of 10–16 MPa, while polyethylene (PE) has a value of around 30 MPa.^71−73^ A comparison of these values with our data suggests that our CVGP material has a superior tensile strength to that of some conventional plastics on the market. The EAB values reported are similar to those reported by Sangsuwan et al. (4.05–4.4%).^70,74^ These values, and hence the flexibility, may be improved by reducing the cross-linker content or altering the plasticizer content.

#### 3.2.2 Moisture Content, Water Solubility, and Water Contact Angle

The water solubility of some biopolymers limits their use as food packaging materials due to potential solubilization and loss of mechanical and barrier properties, leading to the unsuccessful protection of food. Furthermore, many natural biopolymers, including chitosan, are prone to swelling via the absorption of water. Cross-linking techniques are used to overcome this property in this present study. We observed negligible solubilization and a low swelling tendency of the cross-linked films CVGP in water over 4 weeks, reporting a total mass loss of 15.1%. Moreover, we report a moisture content of our CVGP film of 10.1 ± 0.6%, correlating with other chitosan composites reported in the literature.^27,75,76^ Correlating with our findings, Tomadoni et al. reported 20.6% total soluble matter for their CVG film.^33^ Moreover, da Silva et al. reported a reduction in the swelling degree of films with an increased vanillin content.^58^ Souza et al. reported that chitosan films incorporated with natural antioxidant extracts had higher water solubility than pure chitosan films due to the interactions between water, chitosan, and polyphenols.^77^ Indeed, the chitosan polymer usually interacts with water via free hydroxyl and amine groups. However, since these groups are consumed by covalent cross-linking with vanillin in our study, we can assume that they are not available for interaction with water in the CVGP film.

**Figure 2:**
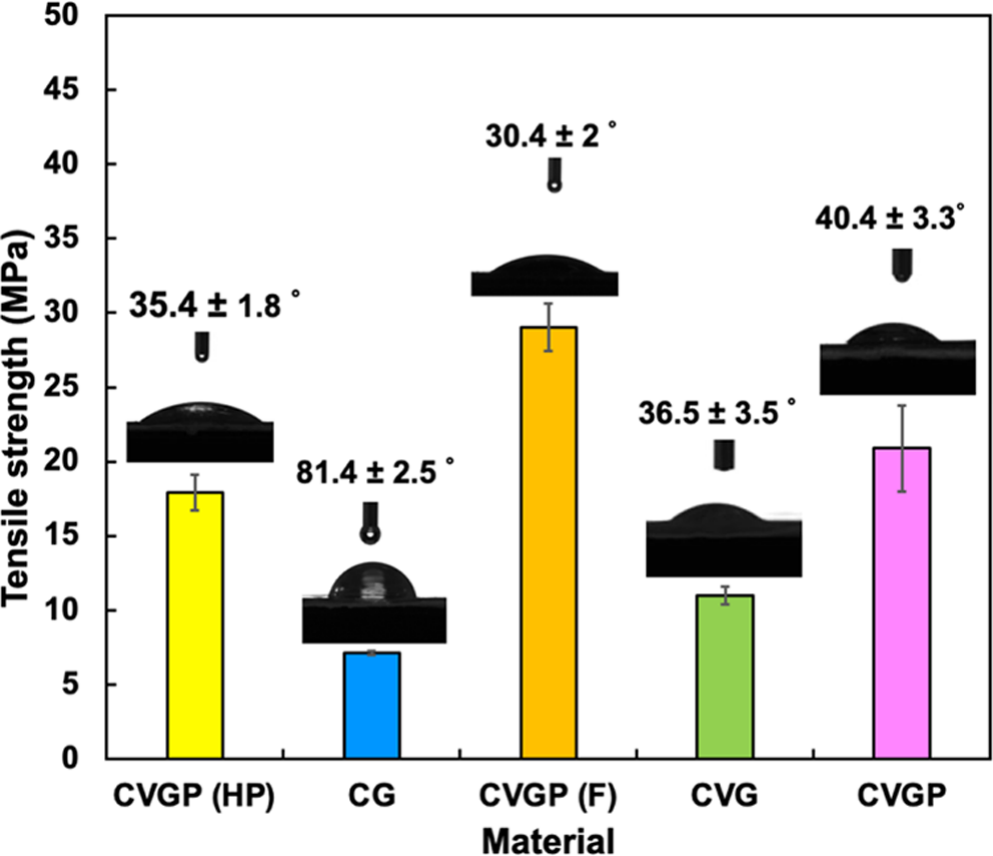
Tensile strength and optical contact angle values of various chitosan-based biopolymer films.

The water contact angle (WCA) is an important index that indicates the hydrophilicity and wettability of films. Hydrophobic packaging materials with high WCA values are more suitable for food with higher moisture contents. Figure 2 shows the contact angle and tensile strength values of the films investigated. The CG films are hydrophobic (84.6°), suggesting strong hydrogen bonds between amino groups in chitosan molecules and an absence of polar functional groups capable of hydrogen bonding on the surface of the film. We hypothesize that the hydroxyl groups of glycerol may interact strongly via hydrogen bonding with chitosan functional groups in non-cross-linked samples, leaving the carbon backbone at the surface. Consistent with the literature, it is expected that the contact angle of CG would decrease over time due to absorption and swelling of the polymer.^78^

Contrastingly, the cross-linked CVG films are hydrophilic in nature (36.5°). Indeed, cross-linking chitosan with glutaraldehyde has been reported to reduce the contact angle of the film.^79^ Another study reported that chitosan–citric acid composites had an initial contact angle of around 65°, which reduced over time.^80^ The group attributed this effect to the evaporation of water and absorption into the polymer matrix.^80^ There is no increase in the hydrophilicity between CVG and CVGP (40.4°), suggesting that the polar moieties of GTE are involved in hydrogen bonding (Figure 2). Alternatively, this may be due to a lack of GTE on the surface of the film, with most of the active components encapsulated within the chitosan–vanillin matrix. Additionally, we concluded that thermal treatments also increased the hydrophilicity of CVGP films. Overall, the results from water sensitivity studies on our CVGP material show a vast decrease in swelling capacity and water absorption of chitosan films upon cross-linking. The results can be related to the success of controlled release studies into food simulants, deducing that the films do not swell or dissolve to a large degree and GTE is likely to be encapsulated within the matrix, able to diffuse out of the film over time (Section 3.1).

#### 3.2.3 Thermal Properties of CVGP Films

**Figure 3:**
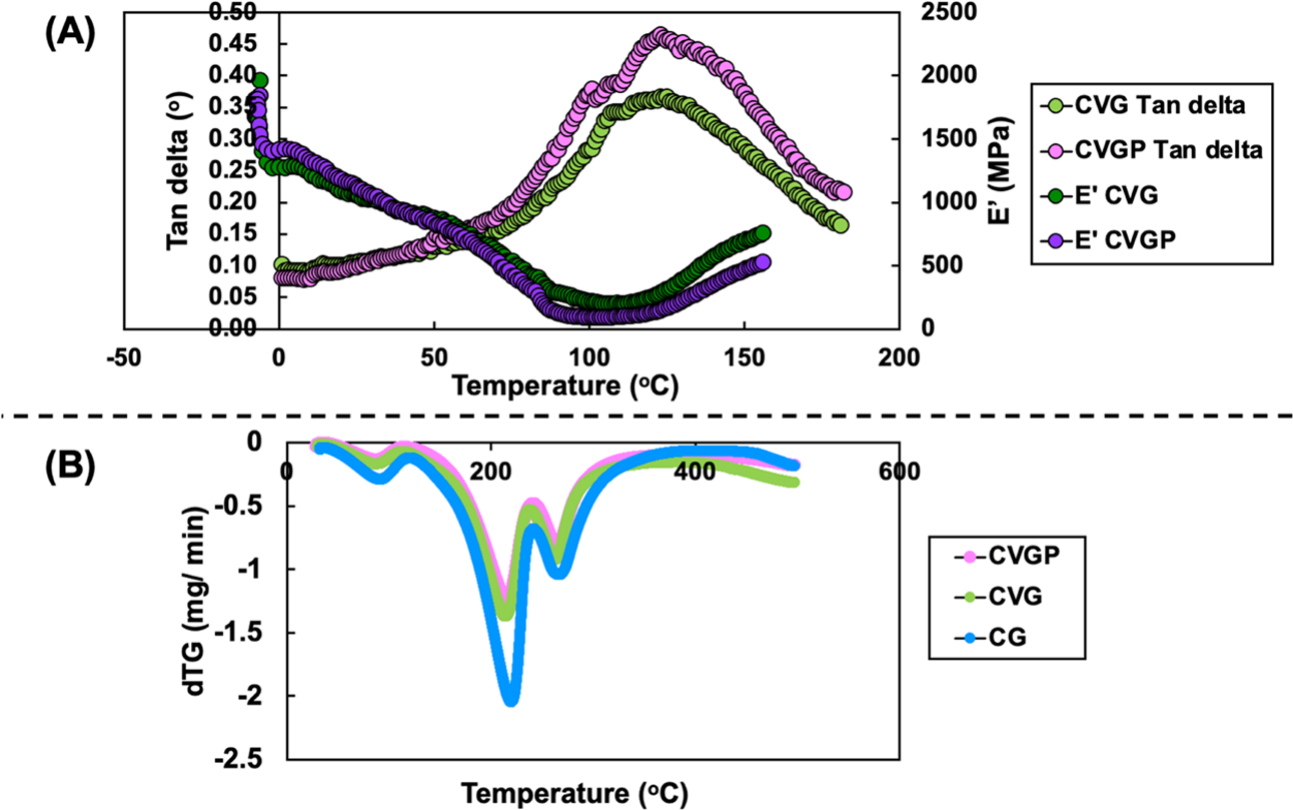
Thermal properties of chitosan-based biopolymeric films: (A) DMAT analysis and (B) TGA analysis.

The thermal properties of biopolymers for packaging materials are important, as they provide an indication of their processability, potential manufacturing processes, and applications. Furthermore, the low thermal conductivity, or insulation provided by thermoplastics or thermoset polymers, helps to protect food from changes to temperature in the environment, thus maintaining quality.^81^ The glass transition temperature (*T*_g_) and the melting temperature (*T*_m_) are important metrics used to indicate the thermal use limits of polymeric materials. In this study, dynamic mechanical analysis was used to study the viscoelastic properties of polymers. The extension loss factor (tan delta) is plotted against temperature to show the index of viscoelasticity (Figure 3A). The maximum value of this plot is the glass transition temperature, which was determined as 123 °C for CVG, 126 °C for CVGP, and 105 °C for CG (Figure S3 in the Supporting Information). The *T*_g_ values obtained suggest an increase in intermolecular and intramolecular interactions in the order of CG < CVG < CVGP. The storage modulus (*E*′) was also reported. This value increases with the strength of intermolecular interactions, showing the stored elastic energy. We found that the *E*′ value decreased with increasing temperature to 125 °C for CVG and CVGP, where it began to increase with temperature (Figure 3A). Here, the increase may be due to further cross-linking interactions induced at higher temperatures.

Thermogravimetric analysis (TGA) was used to measure the thermal stability of chitosan films (Figure 3B). The initial mass loss up to 115 °C is related to the loss of water by evaporation. From these data, we found that the chitosan composites had water contents of 5.1% for CG, 2.5% for CVG, and 3% for CVGP. This result indicates that the cross-linked films retained less water and had lower affinity to water, as indicated in Section 3.2.2. The first degradation stage, between 116 and 129 °C, incurred a mass loss of 43% for CG, 34.5% for CVG, and 34% for CVGP (Figure S4 in the Supporting Information). We report that the decomposition of the chitosan polymer and other film components began at 240 °C. Between 24 and 26%, mass was lost from each film up to 390 °C. We found that the maximum rate of mass loss (*T*_max_) was 220 °C for CG, 216 °C for CVG, and 215 °C for CVGP. The reading continued up to 500 °C, at which point the remaining mass was 22.5% for CVGP. It is expected that the remaining char will decompose between 500 and 700 °C. Similarly, Tomadoni et al. reported two degradation stages: the first included the loss of water bound or absorbed on the polymer (119–146 °C), and the second was related to polymer decomposition (271–282 °C).^33^

### 3.3 Release Studies and Kinetic Analysis

Cross-linking may be used as a technique to promote the controlled release of active compounds from within films, avoiding excessive release due to dissolution breakage or polymer swelling.^30,31^ The release of antimicrobials or antioxidants from thin films is often reported to be governed by random molecular diffusion driven by a concentration gradient. An initial burst release effect due to the surface level or surface-adsorbed molecules is common in most studies. However, many factors affect the mechanism of release including temperature, polymer molecular weight, pH, humidity, film thickness, degradation time frames, and the binding strength between the active agent and the polymer matrix.^71^ For example, lower *M*_w_ chitosan has been reported to enhance release due to weaker binding interactions with the active compound.^71,82^ Moreover, the degradation properties of polymers are particularly important. Oligomers may be released during the release process, changing the porosity of the film over time and opening diffusion channels. Furthermore, the food simulant, which is often a mixture of ethanol and water in varying percentages, can have a profound effect on the release profile depending on the polarity of the active compound.^7^

**Figure 4:**
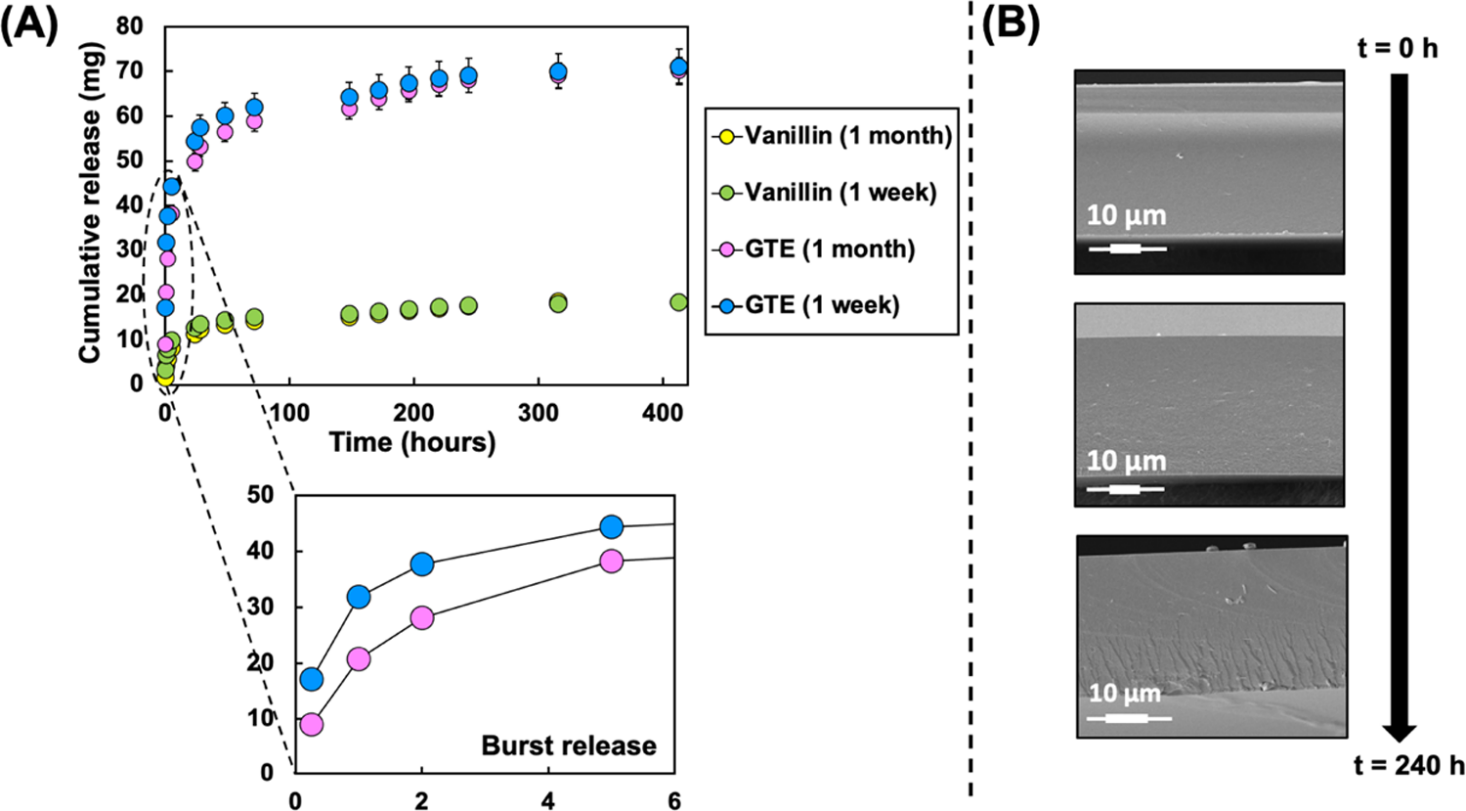
(A) Release profile of polyphenols and vanillin from the CVGP film into a 50% (v/v) ethanol–water food simulant after (i) 1 week storage and (ii) 1 month storage, showing an initial burst release followed by controlled release; the surface area for release was a 4 × 4 cm piece of film with 65 μm thickness. (B) Field emission scanning electron microscopy (FE-SEM) microstructure images of CVGP films at various intervals of submersion in the simulant, showing a uniform surface of the film with increasing irregularity over time.

We investigated the effect of storage time by measuring and comparing the release profiles of films that had been stored in a desiccator for 1 week and 1 month (Figure 4A). In this study, the food simulant of 50% (v/v) ethanol–water was used to mimic amphiphilic foods. As expected, we observed a burst release within the first 8 h, followed by controlled release, which formed a plateau at 48 h. The majority (70%) of the active agent was released within the first 24 h. Since the release is governed by the concentration gradient and the size of diffusion channels, it was expected that the release rate at 8–24 h would be greater than >24 h due to a larger concentration gradient. We can relate this release profile to a mechanism of initial surface, or near-surface, release followed by diffusion-controlled release from within the polymeric network. Vanillin was also released, to a reduced degree, following the same trend (Figure S5 in the Supporting Information). The release of vanillin, assumed to be a minimal proportion of the total content, may be attributed to an excess of vanillin that is not involved in cross-linking. Alternatively, we postulate that water diffusing into the film matrix may interact and exchange with the Schiff base to release vanillin. Importantly, we determined that there was no significant difference in the release profiles of active components between different storage times of the films tested (*p* > 0.05). This suggested that the CVGP films could be stored in a controlled desiccator environment before use for an extended period without losing efficacy.

The SEM images show that there is a visual change in the film over 10 days, whereby the edges are less smooth after 10 days, indicating potential diffusion channels (Figure 4B). During the experiment, the films darkened in color from yellow to orange, which we may attribute to polyphenol oxidation. When applied to food, it is expected that the migration rate would be reduced due to the reduction in water diffusion into the matrix. Indeed, it will be important to determine if the release will be onto the food surface (direct contact), into the headspace (indirect contact) of the packaging, or into the atmosphere. While nonmigrating active compounds may still prevent bacterial growth, we expect that direct contact with high-moisture-content foods may enhance the release of active polyphenols from our CVGP film.

#### 3.3.1 Mathematical Modeling

Important kinetic and mathematical models for active component release studies include zero-order kinetics, first-order kinetics, Fick’s law, and Korsmeyer–Peppas (KP), Hixson–Crowell, Kopcha, and Higuchi methods. Fick’s law is one of the most used single-compartment models for determining diffusion coefficients in the literature. Fick’s law describes the drug release from a thin polymer film and is characterized by an initial *t*_1/2_ dependence of the drug released.^3^ Usually, an initial release (burst release) is followed by a controlled release. Mechanistically, a recent study that used Fick’s second law to fit their initial release data noted that the solvent molecules of the simulant adsorbed to the film surface and permeated into the polymer network over 48 h.^7^ In this study, we will not consider two-compartment mathematical models, since the degradation of CVGP is assumed to be negligible during the release time scale.^83^

**Figure 5:**
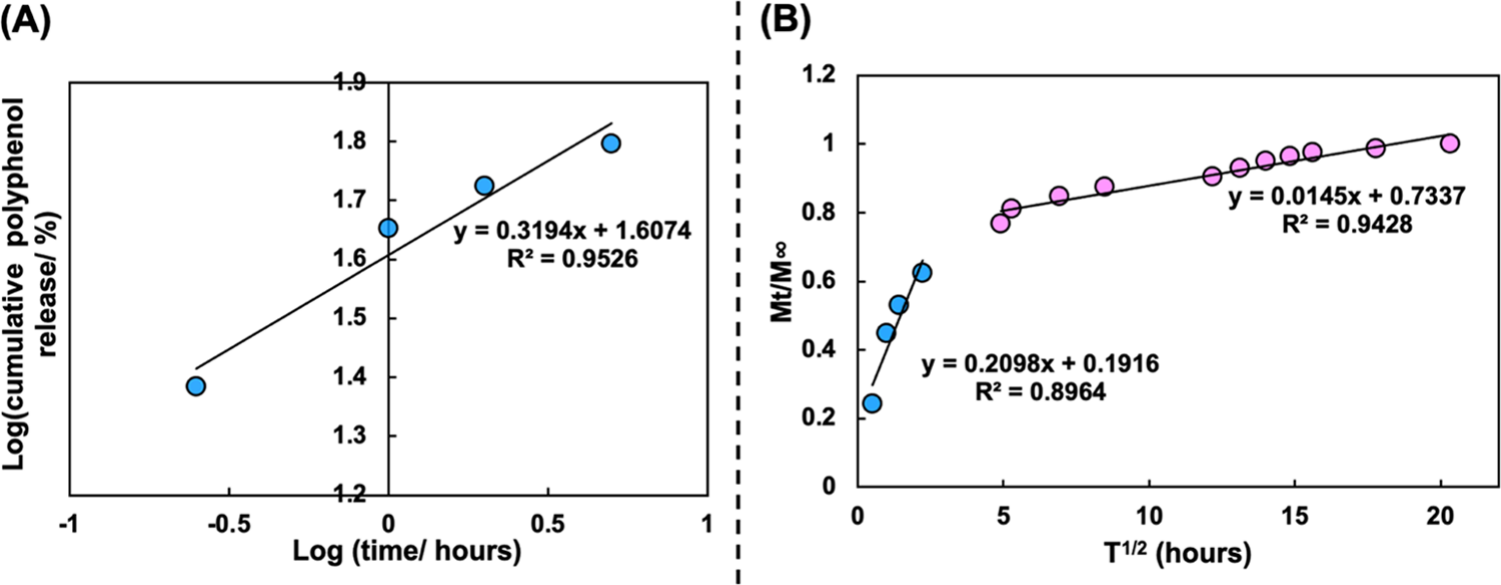
(A) Korsmeyer–Peppas mathematical model for the release of polyphenols from the CVGP cross-linked film. (B) Higuchi model with burst release modeling shown in blue and controlled release shown in pink.

Our release data were initially fit to zero-order kinetics (*R*^2^ = 0.550) and first-order kinetics (*R*^2^ = 0.420) with low correlation values (Figure S6 in the Supporting Information). Therefore, we concluded that these models did not describe the kinetics of the release data recorded. The KP mathematical model describes the release of drugs from a hydrophilic, erodible matrix that may swell with water diffusion. This model was used to fit the raw data (Figure 5A) for the first 60% of the release. Using this KP plot, the release constant (*K*_KP_) was determined as 41 h^–*n*^. The “*n”* value describes the mechanism of release, with a value of ≤0.45 indicating Fickian diffusion. The value of *‘n’* in this case was determined as 0.32 using eq 5, suggesting a Fickian mechanism. CVGP is hydrophilic and erodible, satisfying the criteria of the KP model. However, during the release time scale, we can assume minimal erosion and swelling (Sections 3.2.2 and 3.4). We also applied the Higuchi model, which describes drug release through an insoluble thin film matrix via a plot of cumulative release (%) against *t*_1/2_ (hours; Figure 5B). The release data were fit to two linear Higuchi models, one to describe the initial release and the second to describe the controlled release mechanism. This method was utilized as a simplification of the nonlinear data set by treating the two phases of release separately. The release constant *K*_H_ was determined as 0.21 h^–1/2^ for the initial release and 0.0015 h^–1/2^ for the controlled release. Additionally, using an approximation derived from Fick’s second law, it was possible to obtain the diffusion coefficient for the burst release as *D* = 5.542 × 10^–11^ m^2^ s^–1^ using Figure 5B and eq 8. We can consider this value as the maximum rate of release with a film thickness of 65 μm (Figure 5B). The kinetic constant *k* for the Fickian approximation was also determined as 0.21 h^–1/2^. Similar release studies using carvacrol as the active compound have reported semi-Fickian diffusion over 80 h, with complete release at 12 h^84^ and first-order kinetics.^82^ Indeed, in our study, we hypothesize that the observed Fickian mass transfer kinetics was governed by the interaction of green tea polyphenols and the chitosan–vanillin matrix.

### 3.4 Antioxidant and Antimicrobial Performance

**Figure 6:**
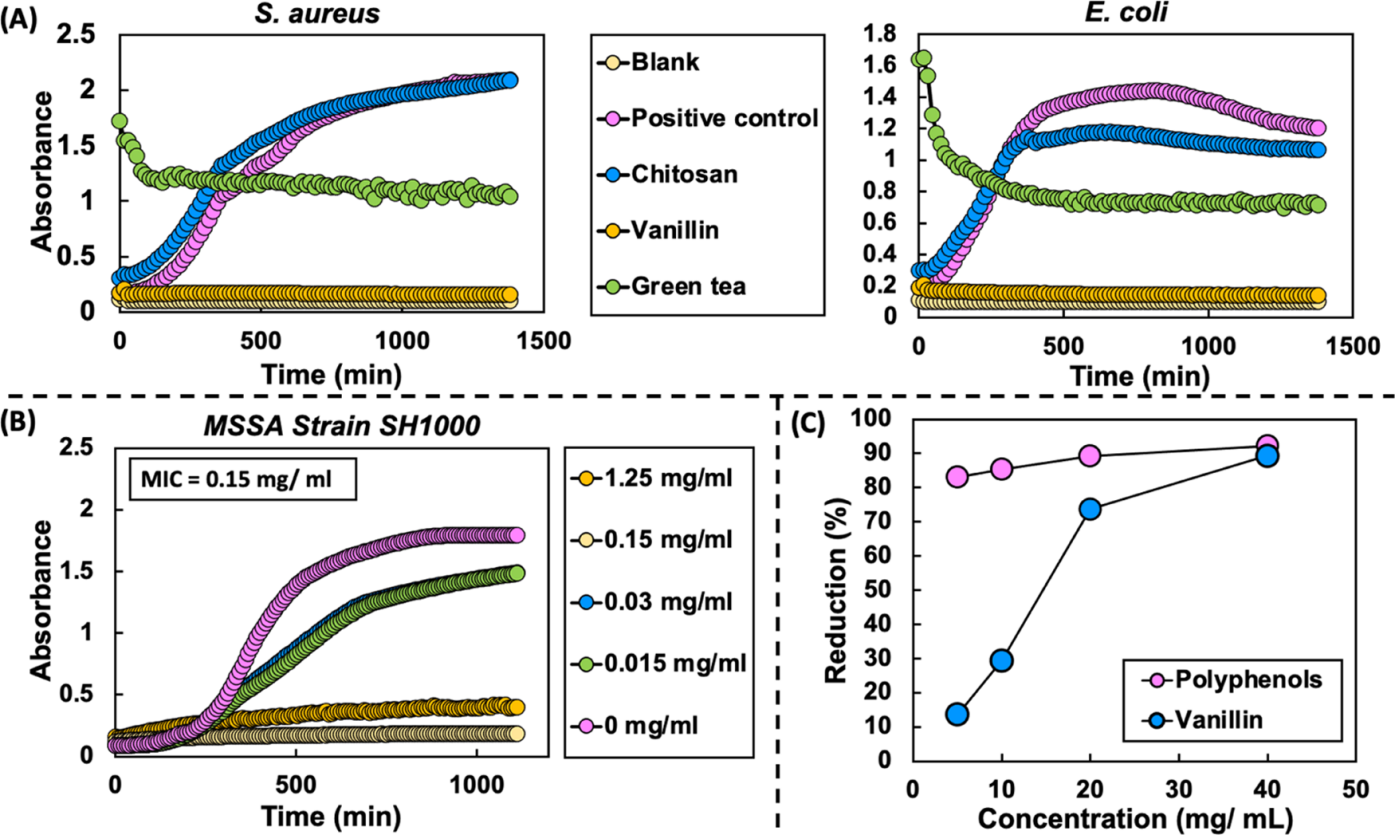
Antimicrobial activity of (A) various components of the active CVGP film against *E. coli* and *S. aureus*, where the positive control is inoculated LB broth. (B) Green tea polyphenols against *S. aureus* bacteria at different concentrations of the extract. GTE absorbance is elevated due to the color of the extract, and the complete data set for MIC determination is reported (Figure S8 in the Supporting Information), (C) The reduction (%) of the DPPH radical against the concentration of the active agents, showing their antioxidant capacity.

The antimicrobial activity of active packaging is a vital characteristic relating to the extension of the food shelf life. Cell counter overnight growth curves were carried out using *E. coli* and *S. aureus* with various film components at 20 mg mL^–1^ concentrations to determine their inhibitory activity (Figure 6A). The results indicate that GTE and vanillin are effective inhibitors of both bacterial strains. Consistently with these results, vanillin has been used as an active compound in various studies, inhibiting food-related bacteria.^35,57,70,85,86^ Chitosan did not show an inhibitory effect in this study. However, chitosan is widely reported as an antibacterial polymer.^87^ Although the exact mechanism of the antimicrobial action of chitosan is not clear, the protonated amino sites have been postulated to alter the permeability of microbial cells via electrostatic interactions with their membranes.^22,88^ Furthermore, the active green tea polyphenol compound was exposed to a strain of *S. aureus* strains at varying concentrations to determine the MIC as 0.15 mg mL^–1^ (Figure 6B). This MIC value can be related to the release study, where the release amounted to over 7 mg mL^–1^ (Figure 5A). Therefore, this result may be considered as a safe-level, effective antimicrobial strength. These results correlate with those reported in a literature study describing the antibacterial effect of chitosan–GTE films against foodborne bacteria.^89^ Moreover, the MIC determined for GTE in this study can be compared to a study that reported an MIC of their green tea extract as 0.4 mg/mL against *S. aureus* ATCC 25923.^90^ Additionally, gar “zone of inhibition tests” were also carried out on CG, CVG, and CVGP film-forming solutions, qualitatively showing the successful inhibition of the bacterial growth of both *E. coli* and *S. aureus* (Figure S7 in the Supporting Information).

Many natural compounds contain antioxidant phenolic moieties that elicit strong radical scavenging capacities. To provide an insight into the release of antioxidant constituents, we performed a DPPH assay. Indeed, our results show strong antioxidant capacities of both vanillin and green tea extract, alluding to a potential synergetic effect (Figure 6). From the data, GTE has an antioxidant activity of >80% at each concentration tested, while vanillin reached >70% inhibition at 30 mg mL^–1^. Tomadoni et al. reported the DPPH inhibition of their CVG film as 45%.^33^ We can relate these values to the release profile discussed in Section 3.3 (Figure 4A). For the release study, a small section of the film was used, and extrapolating the effect to the size required for packaging would exceed the concentration values reported for good antioxidant activity. Various studies have highlighted the potential of active films to inhibit viral replication.^91^ We carried out an initial investigation into the antiviral capacity of the film. The data revealed that there was a >50% reduction in the amount of virus when CVGP films were used as the testing surface (Figure S9 in the Supporting Information). Viral inhibition is observed, however the results are not conclusive of strong antiviral activity due to low log-removal values.

### 3.5 Degradation Behavior

The degradation of polymers depends on the polymer chain length, molecular weight, the complexity of the chemical formula, and the crystallinity of the polymer.^92^ Examples of industrial test methods to determine degradability include biodegradation tests (ISO 14855) and disintegration tests (ISO 16929), with a maximum testing duration of 180 days within a specified temperature range. “Biodegradable” polymers have a primary degradation mechanism based on microorganism-related metabolism.^93^ Pristine chitosan has a good biodegradation capacity, which may be enhanced using ubiquitous hydrolase enzymes.^22^ Importantly, current legislation states that the degree of disintegration at 12 weeks must be greater than or equal to 90% to term a material as “compostable”.^94^

**Figure 7:**
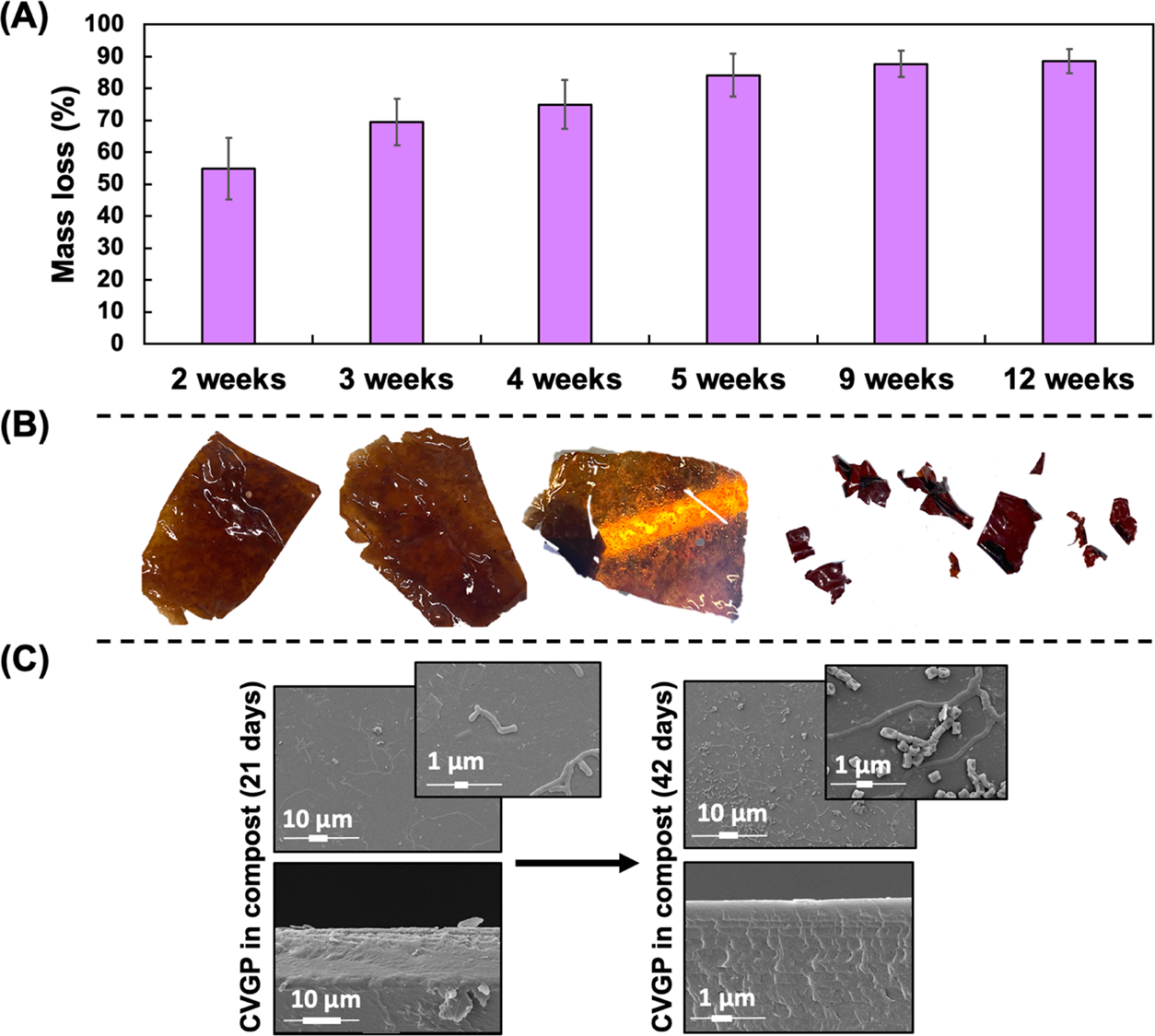
(A) Dry weight loss tests of CVGP films over 12 weeks of degradation in compost, (B) images of CVGP films at varying stages of degradation, and (C) corresponding SEM images of the films.

We buried CVGP films in commercial compost and measured the remaining mass of the films at regular intervals for up to 12 weeks; water was added when required to maintain a constant moisture content and good conditions for bacterial growth. We report a promising degradability of 91.3 ± 2.3% by weight loss for CVGP films (Figure 7A). Visually, the films darkened, became fragile, and broke into smaller fragments during degradation (Figure 7B). The SEM images at 3 and 6 weeks show structural film changes due to degradation (Figure 7C). We also carried out degradation investigations using soil from the University of Bath campus and seawater from Devon. We report that CVGP samples degraded to 59.8 ± 2% in soil over 12 weeks and 50.6 ± 1.5% in seawater over 8 weeks (Figure S10 in the Supporting Information). The degradation in these environments was slower than the degradation in compost, likely due to a lower number of bacteria and microorganisms in these samples. While pristine chitosan films have been reported to degrade completely in a variety of soil types.^95^ Previous studies of vanillin cross-linked chitosan have not incorporated an examination of the degradation of the material. One literature study on a composite film of chitosan–PCL–grapefruit seed extract reported a dry weight loss of 27% over 16 weeks.^71^ Moreover, Altun et al. reported the CO_2_ emission data for chitosan degradation in reactors inoculated with microorganisms, concluding that microorganisms capable of producing chitosanase enzymes improved the efficiency of the degradation process.^96^ Overall, these results indicate that the material is promising in terms of compliance with degradation legislation, although further tests are required to confirm this property.

In conclusion, the results reported here demonstrate the fabrication and analysis of vanillin cross-linked films with a controlled release of green tea polyphenols for active food packaging. The chemical FTIR data confirmed that the cross-linking method was mediated by the formation of a Schiff base between vanillin and chitosan, confirmed by a shift in the peaks from 1656 to 1644 cm^–1^. The optical properties of the film showed that cross-linking enhanced the yellow color of the film. The moisture content of the CVGP film was determined as 10.1 ± 0.6%, and the total soluble matter was determined as 15.1%. The CVGP films were hydrophilic with a water contact angle of 40.4 ± 3.3°. However, due to optimized cross-linking, thin CVGP films did not form hydrogel materials upon addition to water. Additionally, cross-linking chitosan with vanillin increased the tensile strength (20.9 ± 2.9 MPa) and the glass transition temperature (126 °C) for CVGP films. The release studies performed described a burst release effect followed by the controlled release of green tea polyphenols for a testing period of 400 h. We found that the amount released exceeded the MIC values determined during antimicrobial testing (0.15 mg mL^–1^, *S. aureus*). Kinetic analysis of the release data followed a Fickian initial release, and Korsmeyer–Peppas and Higuchi models were also applied to the data to obtain corresponding release constants. Both vanillin and green tea extract showed strong antibacterial and antioxidant activities, with all concentrations of polyphenols tested quenching >80% of DPPH radicals. Furthermore, the CVGP film showed promising initial degradability (>85%) within 12 weeks in compost. Overall, these tests suggest that our material may be used to package foods with low acidity levels and various moisture contents. We postulate that high-moisture-content foods such as fruits and vegetables may trigger the controlled release of green tea polyphenols and enhance the antibacterial effect. However, the material would also be applicable to low-moisture-content foods such as nuts, dry products, and oily foods. In conclusion, our results highlight the potential application of this novel material in the food packaging industry.

## Supporting Information Available

UV–visible calibration curves for green tea polyphenols and vanillin (Figure S1); schematic of Schiff base formation (Figure S2); DMAT analysis of CG films (Figure S3); TGA analysis of CG, CVG, and CVGP films (Figure S4); data for vanillin release from CVGP films (Figure S5); zero- and first-order mathematical fitting of release data (Figure S6); agar bacterial inhibition images of polyphenols and film-forming solutions against MRSA, *E. coli* and *S. aureus* (Figure S7); full antibacterial data for MIC determination (Figure S8); antiviral inhibition of *P. syringae* by chitosan-based films (Figure S9); and degradation data for CVGP films in soil and seawater (Figure S10) (PDF)
