# Supplementary material for: Vanillin Cross-Linked Chitosan Film with Controlled Release of Green Tea Polyphenols for Active Food Packaging
Source: ACS Food Sci Technol. 2023 Oct 9;3(10):1680–93. doi: 10.1021/acsfoodscitech.3c00222 (PMC10594654; doi:10.1021/acsfoodscitech.3c00222)
Supplement: Supplementary file 1 — fs3c00222_si_001.pdf [file fs3c00222_si_001.pdf]

Vanillin cross-linked chitosan film with controlled release of green tea polyphenols for active food packaging

Submitted to

ACS Food Science and Technology

Date: May 2023

Jessica R. Westlake †, Maisem Laabei ◊, Yunhong Jiang ¥, Wen Chyin Yew ¥, Darren L. Smith ¥, Andrew D. Burrows ‡, Ming Xie †

†Department of Chemical Engineering, University of Bath, Bath, BA2 7AY, UK.

‡Department of Chemistry, University of Bath, Bath, BA2 7AY, UK.

◊Department of Biology, University of Bath, Bath, BA2 7AY, UK.

¥Department of Applied Sciences, Northumbria University, Newcastle, NE7 7XA, UK.

Corresponding authors:

Ming Xie, mx406@bath.ac.uk

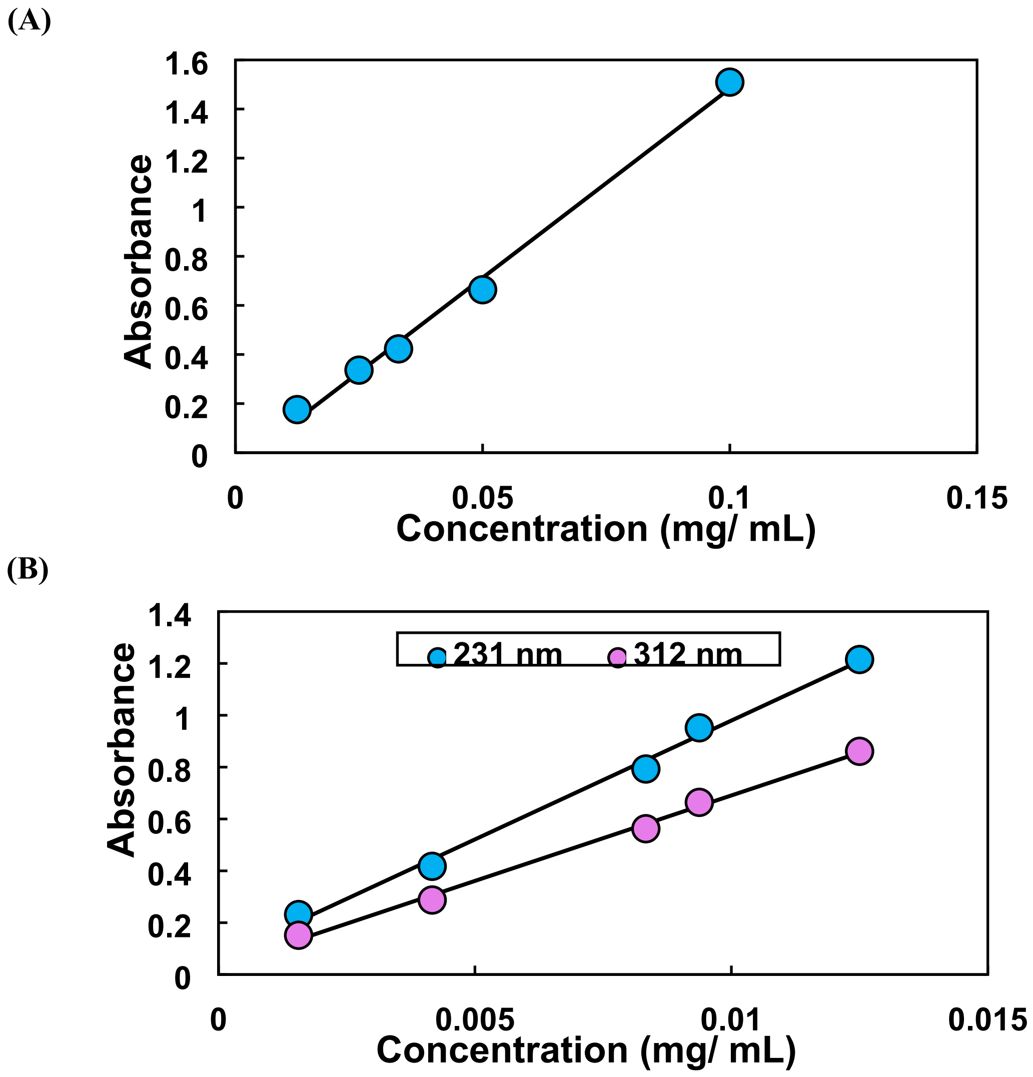

Figure S1. UV-Visible concentration calibration curves for green tea polyphenols (A) and vanillin (B) in 50% (v/v) ethanol-water food simulant

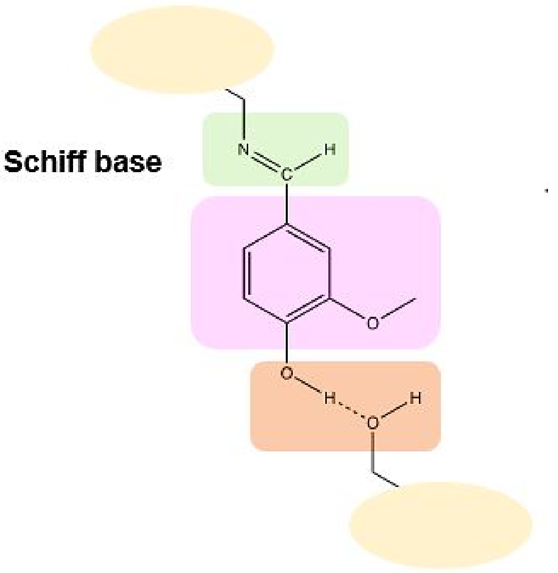

Figure S2. Schematic of Schiff base formation and proposed cross-linking mechanism between vanillin and chitosan chains (represented in yellow)

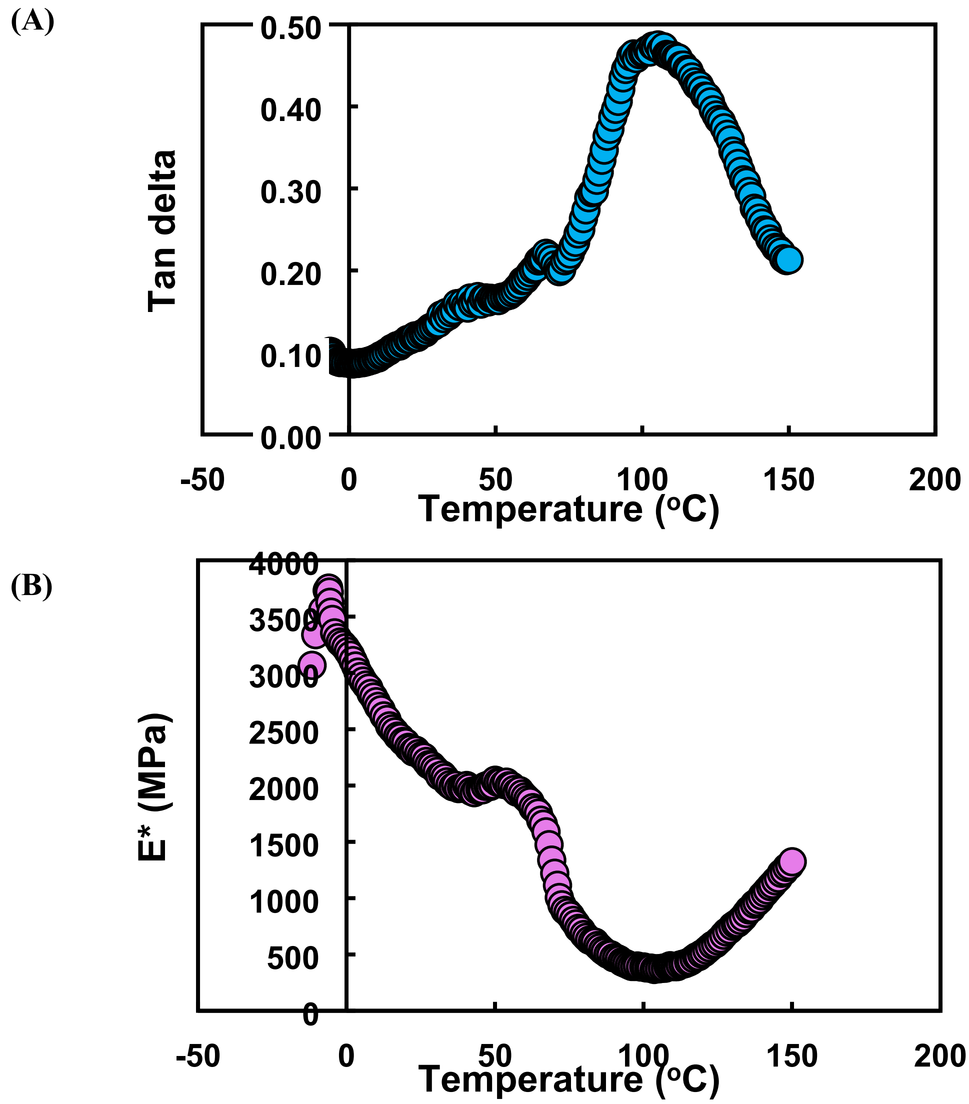

Figure S3. DMAT analysis of CG film (A) extension loss factor plot of tan delta, (B) storage modulus plot of E*

(A)

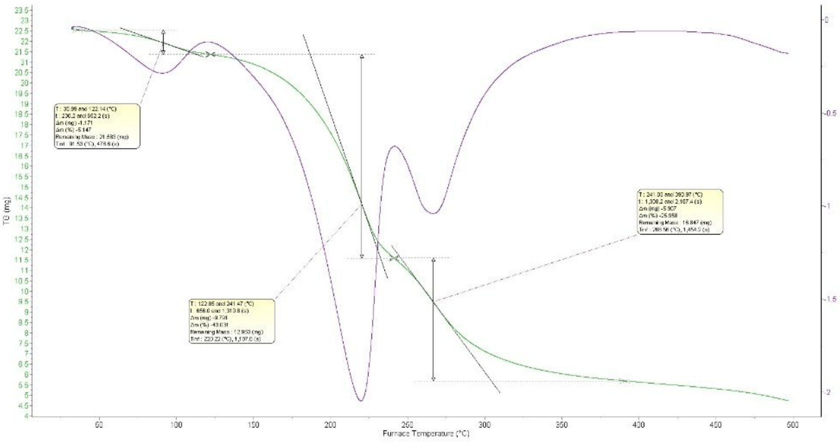

(B)

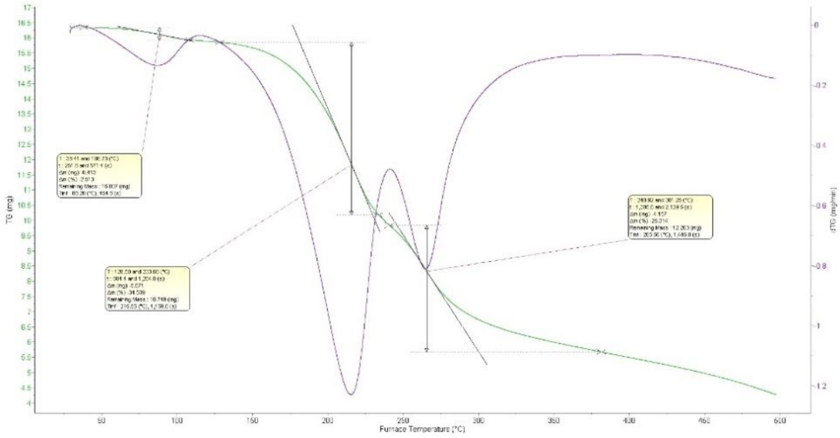

(C)

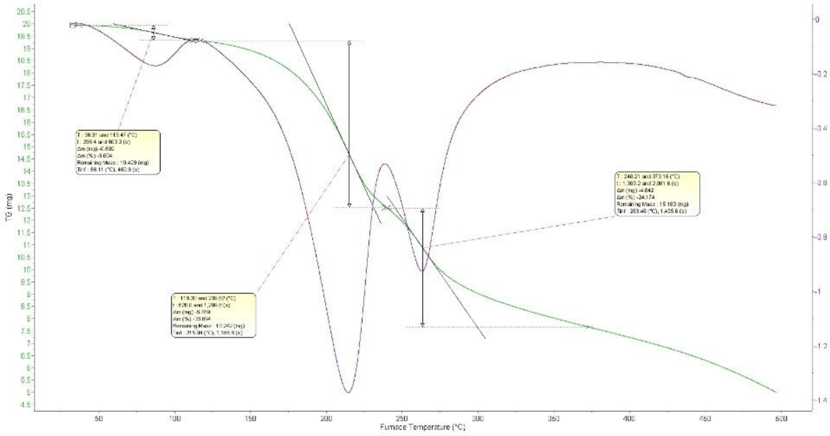

Figure S4. Raw TGA data analysis and derivation of percentage mass changes for CG (A), CVG (B), and CVGP (C)

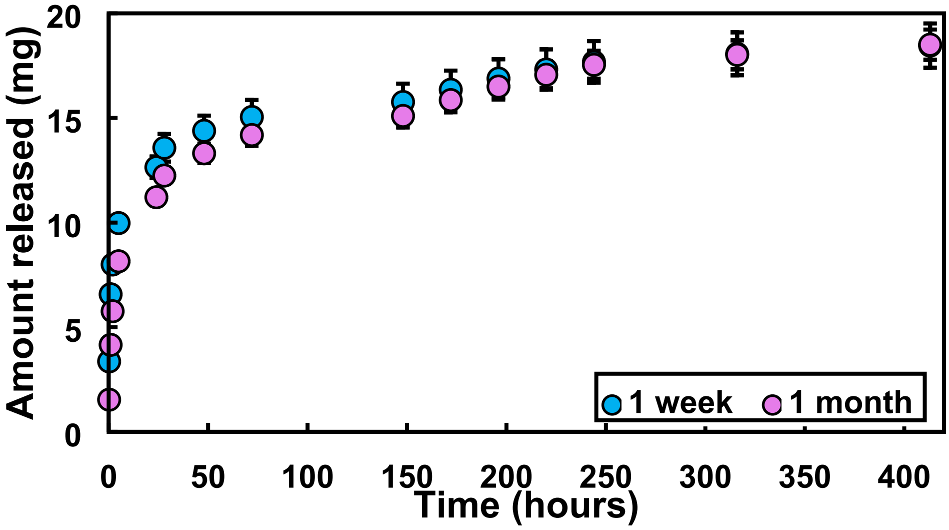

Figure S5. Vanillin release from CVGP film into 50% (v/v) ethanol-water simulant

(A)

(B)

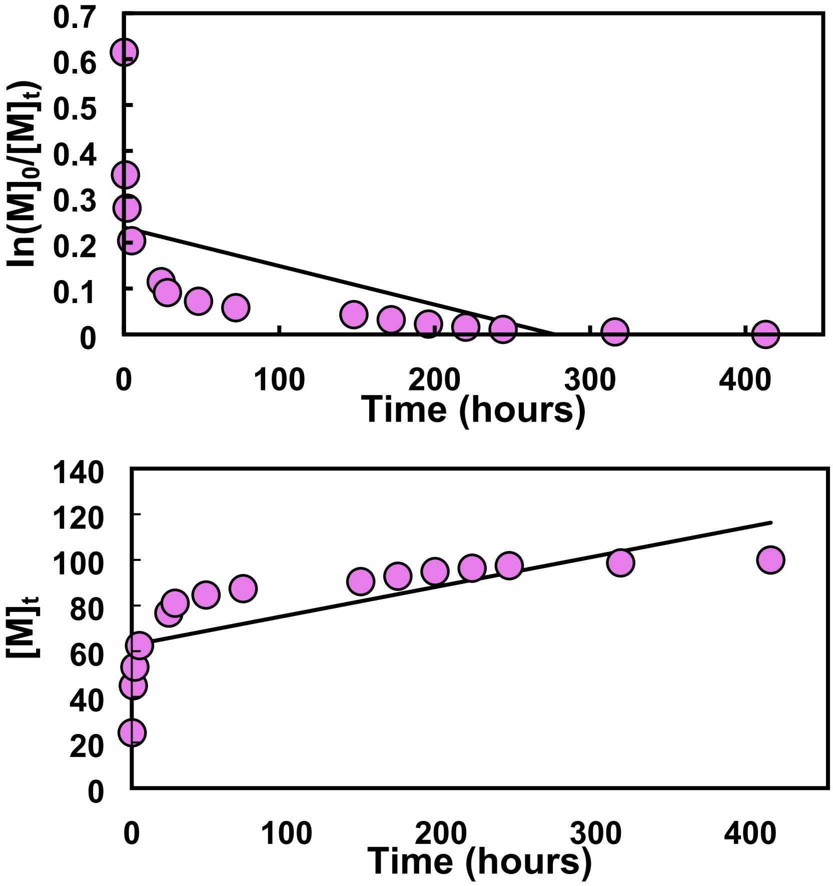

Figure S6. First-order (A) and zero-order (B) mathematical fitting of release kinetics of green tea polyphenols

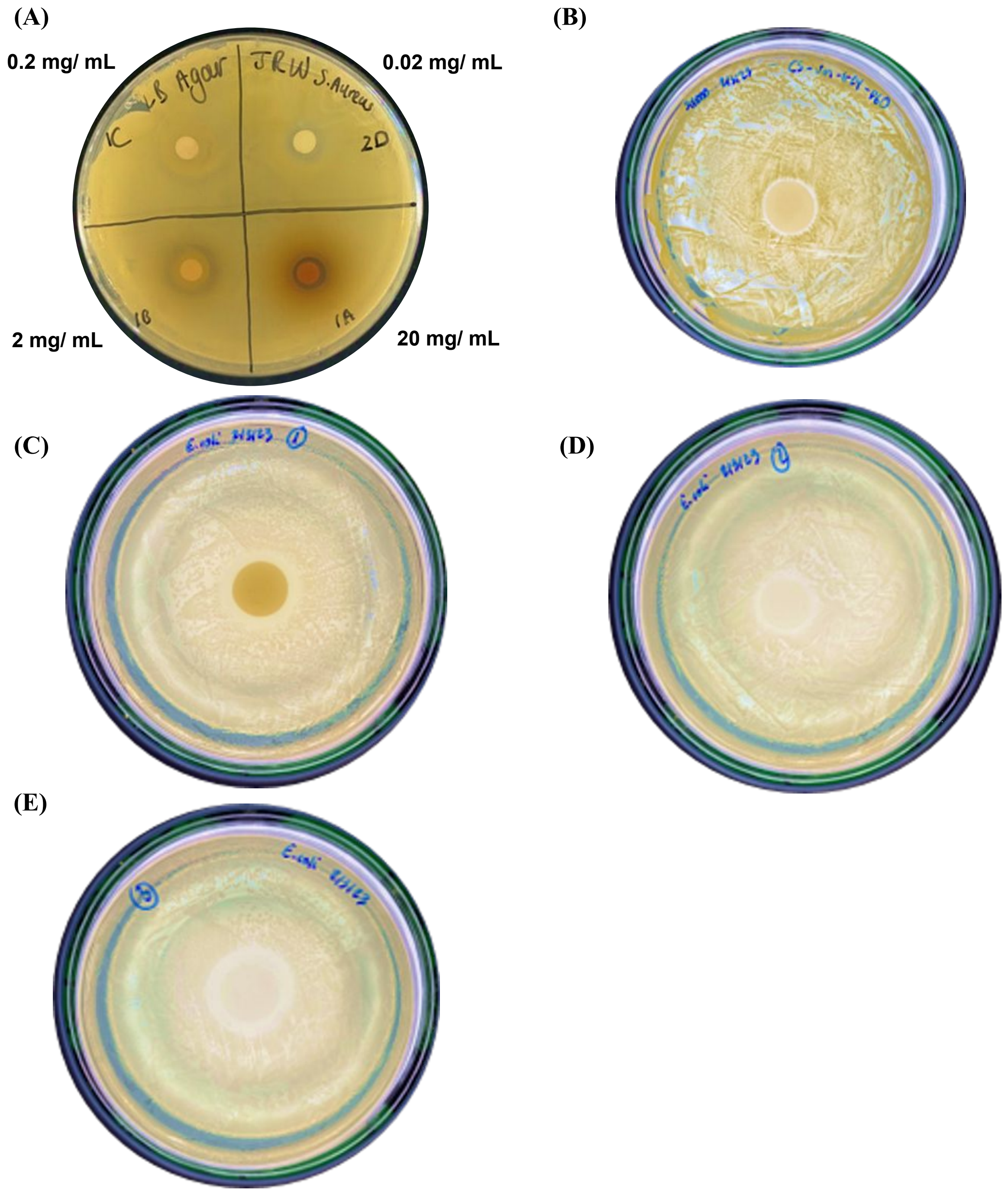

Figure S7. Agar plates showing bacterial inhibition of film forming solutions and components (A) disc-diffusion assay of polyphenols against *S. aureus* with varying concentration (B) CVGP film forming solution against *S. aureus*, (C) CVGP against *E. coli*, (D) CVG against *E. coli*, (E) CG against *E. coli*

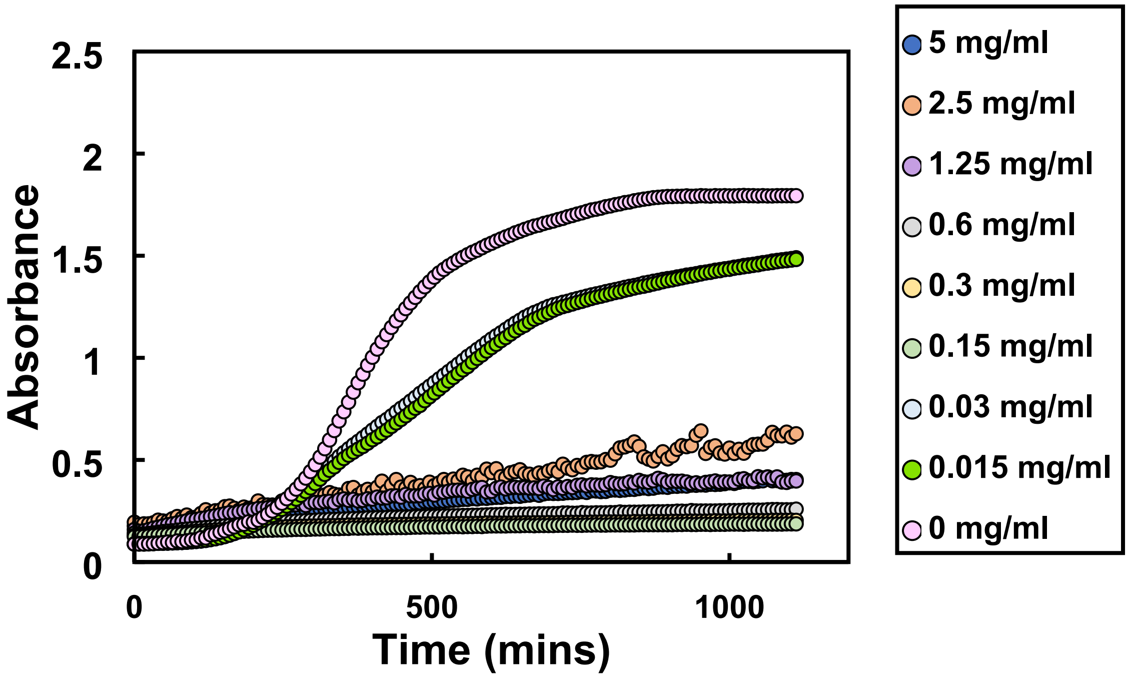

Figure S8. MIC determination of green tea polyphenols against *S. aureus* bacteria at different concentrations of the extract

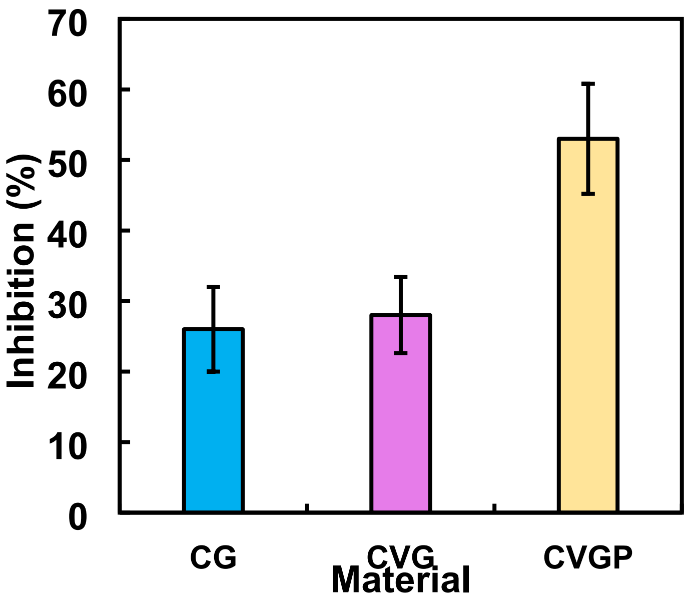

Figure S9. Antiviral inhibition of three chitosan films against *P. syringae*

(A)

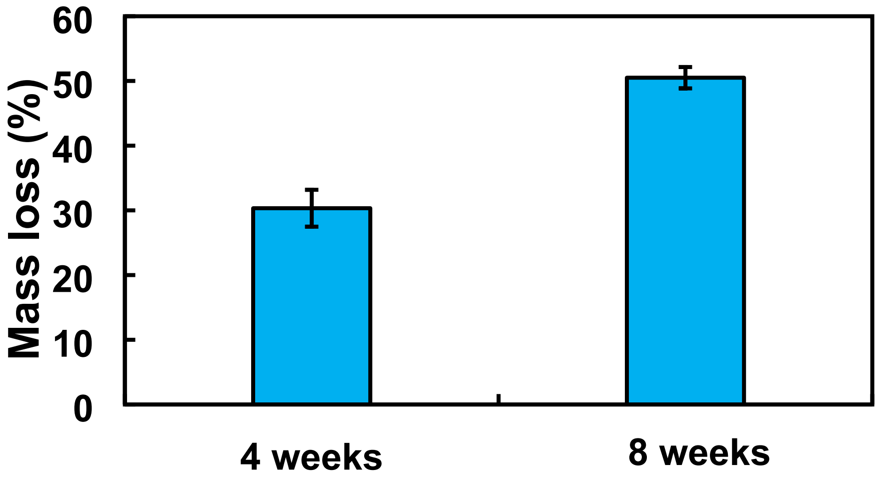

(B)

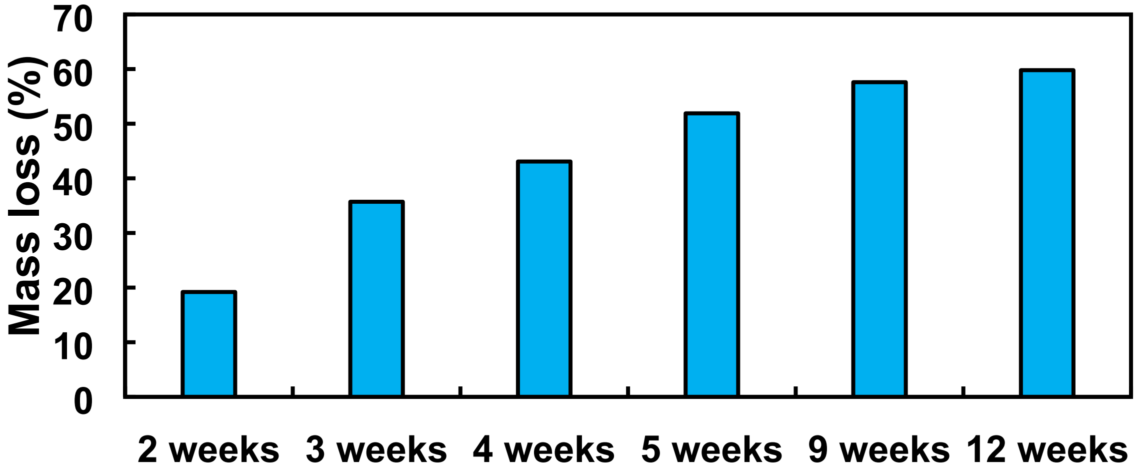

Figure S10. Degradation of (A) CVGP in seawater over 8 weeks and (B) CVGP in soil over 12 weeks
